# Supplementary material for: Content-rich biological network constructed by mining PubMed abstracts
Source: BMC Bioinformatics. 2004 Oct 8;5:147. doi: 10.1186/1471-2105-5-147 (PMC528731; doi:10.1186/1471-2105-5-147)
Supplement: Additional File 2 — The original results of the above study (non-essential files are deleted to keep the file size under the limit set by BMC bioinformatics). [file 1471-2105-5-147-S2.bz2 › chilibotAdditionalFile2/dip20/154ID7630397E551/html/CDK2_CCNA2.html]

 


 **CDK2** and **CCNA2** 
  
Found 650 abstracts in PubMed, retrieved 20.  
 

 What does Google say? 
 PDF only 
| .edu only 

---

**Interactive relationship** (e.g. stimulation, inhibition, etc)

**Inhibitory relationship**- The inhibition of cyclin A  [ **CCNA2** ]  gene expression is associated with a block in cyclin E  **cdk2**  activity and phosphorylation of both the retinoblastoma protein and p107.  Ref: 12869629 Mol Pharmacol, 2003
**Stimulatory relationship**- The decrease in  **CDK2**  kinase activity was the result of increased p21 association with cyclin A  [ **CCNA2** ]   **CDK2**  and cyclin E  **CDK2** .  Ref: 12810668 Cancer Res, 2003
- However, in both cell types Cdk 2  [ **CDK2** ]  activity associated with cyclin E and cyclin A  [ **CCNA2** ]  showed an increase only when the cells transited from G 1 into S phase.  Ref: 12704643 J Cell Physiol, 2003
**Neutral relationship**- This is a novel mechanism of TGF beta1 inhibition requiring early G 1 induction and stabilization of p21 protein, which binds to and inhibits cyclin E  **CDK2**  and cyclin A  [ **CCNA2** ]   **CDK2**  kinase activity rather than direct modulation of cyclin or CDK protein levels as seen in other systems.  Ref: 12810668 Cancer Res, 2003

**Non-interactive relationship** (e.g. studied together, co-existance, homology, etc.)

- A microscopic study of functional disorder order folding transitions coupled to binding is performed for the p27 protein, which derives a kinetic advantage from the intrinsically disordered unbound form on binding with the phosphorylated cyclin A  [ **CCNA2** ]  cyclin dependent kinase 2  [ **CDK2** ]   **Cdk2**  complex.  Ref: 12697905 Proc Natl Acad Sci U S A, 2003
- The roles of cyclin A  [ **CCNA2** ]  E  **CDK2**  complexes in driving replication, their relationship with other regulators of S phase, and their role in keeping replication to only once per cell cycle will be discussed.  Ref: 12851482 Cell Cycle< MedlineTA>Cell Cycle, 2003
- Adriamycin induced G1 S arrest seems to be realized via cyclin cdk complexes activity independent way involving antiproliferative targets downstream of cyclin E  **cdk2**  and cyclin A  [ **CCNA2** ]   **cdk2**  complexes.  Ref: 12722479 Tsitologiia, 2003
- Cyclin A  [ **CCNA2** ]   **cdk2**  appears to associate with NF Y both in vitro and in vivo.  Ref: 12857729 J Biol ChemJ Biol Chem, 2003
- Similarly, levels of the p21 WAF1 CIP1 protein that is associated with the Cyclin A  [ **CCNA2** ]   **Cdk2**  complex increased, and these increases correlated well with the down regulation of Cyclin A  [ **CCNA2** ]   **Cdk2**  activity.  Ref: 12819186 Carcinogenesis< MedlineTA>Carcinogenesis, 2003
- Arsenic increased expression of the P21 protein and decreased levels of cyclin A  [ **CCNA2** ] , cyclin B1 and cyclin D1, but expression of  **CDK2** , CDK4, CDK6, and cyclin E were not affected.  Ref: 12883267 Pancreas, 2003
- Furthermore, cyclin A  [ **CCNA2** ]  CDK1 2, cyclin B CDK1 2 and cyclin E  **CDK2**  complexes can phosphorylate multiple p73 isoforms in vitro at threonine 86 T86.  Ref: 12676926 J Biol Chem< MedlineTA>J Biol Chem, 2003
- Thus, PD selectively elevates p21 WAF1 CIP1 levels and thereby arrests the cell cycle at G1 S phase by down regulating Cyclin A  [ **CCNA2** ]   **Cdk2**  activity.  Ref: 12819186 Carcinogenesis< MedlineTA>Carcinogenesis, 2003
- In vitro kinase assays demonstrated a corresponding massive activation of the cyclin A  [ **CCNA2** ]  regulated cyclin dependent kinase 2  [ **CDK2** ]   **cdk2**  but not of  **cdk2**  protein level in muscle homogenates after 1.5 days of muscle loading.  Ref: 12819222 J Appl Physiol< MedlineTA>J Appl Physiol, 2003
- Cyclin E  **CDK2**  and cyclin A  [ **CCNA2** ]   **CDK2** , together with DBF4 CDC7, phosphorylate several components of the prereplication complex and replication machinery.  Ref: 12851482 Cell Cycle< MedlineTA>Cell Cycle, 2003
- Monensin decreased the levels of  **CDK2** , CDK6, cdc2, cyclin A  [ **CCNA2** ] , cyclin B1, cyclin D1 and cyclin E proteins but did not alter CDK4 protein.  Ref: 12792794 Int J Oncol, 2003
- Furthermore, YA protein is phosphorylated in parallel with a cell cycle dependent activation of  **cdk2**  kinase and cyclin A  [ **CCNA2** ]  expression.  Ref: 12857729 J Biol ChemJ Biol Chem, 2003
- Panaxadiol selectively inhibits Cyclin A  [ **CCNA2** ]  associated  **Cdk2**  activity by elevating p21WAF1 CIP1 protein levels in mammalian cells.  Ref: 12819186 Carcinogenesis< MedlineTA>Carcinogenesis, 2003
- Positive staining for cyclin D1, cyclin E, cyclin A  [ **CCNA2** ] , cyclin B1,  **cdk2** , cdk4, cdc2, p53, p21, and p27 was observed in 63%, 66%, 31%, 32%, 51%, 77%, 71%, 43%, 35%, and 60% of the 82 carcinomas, respectively.  Ref: 12792921 Hum Pathol, 2003
- it delayed the activity of the cyclin A  [ **CCNA2** ]   **cdk2**  complex to 34h.  Ref: 12876382 Cell Physiol Biochem, 2003
- PD treated cells were arrested at G1 S phase, which coincided well with decreases in Cyclin A  [ **CCNA2** ]   **Cdk2**  activity, but not in Cyclin E  **Cdk2**  and Cdc2 activities.  Ref: 12819186 Carcinogenesis< MedlineTA>Carcinogenesis, 2003
- Western blotting analysis revealed that the protein levels of cyclin A  [ **CCNA2** ] , cyclin E, and cyclin dependent kinase CDK 2  [ **CDK2** ]  but not cyclin D1 and CDK4 decreased after progesterone treatment, but those of CDK inhibitory proteins, p21 and p27, increased.  Ref: 12810531 Endocrinology< MedlineTA>Endocrinology, 2003
- Overexpression of the replication initiation factors Cdt1 and Cdc6 along with cyclin A  [ **CCNA2** ]   **cdk2**  promotes rereplication in human cancer cells with inactive p53 but not in cells with functional p53.  Ref: 12718885 Mol Cell, 2003
- Immunoprecipitation studies demonstrated that Sp1 interacts with cyclin E, cyclin A  [ **CCNA2** ]  and cyclin dependent kinase 2  [ **CDK2** ]  during the S phase.  Ref: 12794070 J Biol Chem< MedlineTA>J Biol Chem, 2003
